# Therapeutic Innovations for Monkeypox Inhibition

**DOI:** 10.3390/ijms27104307

**Published:** 2026-05-12

**Authors:** Nayan De, Jhuma Bhadra, Md Sorique Aziz Momin, Kamala Mitra, Debmalya Bhunia, Achinta Sannigrahi

**Affiliations:** 1 Department of Biomedical Engineering, John A. White, Jr. Engr. Hall Fayetteville, University of Arkansas, Fayetteville, AR 72701, USA; 2 School of Applied & Interdisciplinary Sciences, Indian Association for the Cultivation of Science, Kolkata 700032, India; saisjb@iacs.res.in; 3 Department of Chemistry, Sarojini Naidu College for Women, Kolkata 700028, India; 4 School of Physics and Astronomy, College of Science, Rochester Institute of Technology, Rochester, NY 14623, USA; 5 Department of Chemistry, Prasanta Chandra Mahalanobis Mahavidyalaya, Kolkata 700108, India; 6 Cold Spring Harbor Laboratory, 1 Bungtown Rd., Cold Spring Harbor, NY 11724, USA; 7 University of Texas Southwestern Medical Center, 5323 Harry Hines Blvd, Dallas, TX 75390, USA

**Keywords:** Monkeypox, antiviral, small molecules, CRISPR, peptide nucleic acid, combination therapy, nanotechnology

## Abstract

This review investigates biomaterial-based strategies for improved treatment of MPXV. We focus on emerging synthetic biomedical approaches to combating the virus. These include peptide nucleic acids, CRISPR-based systems, and small-molecule therapeutics. These methods work by targeting and blocking viral proteins and enzymes. Such synthetic platforms may help reduce viral transmission and minimize side effects. They also offer potential solutions to challenges such as viral resistance in humans. In addition, biomaterials contribute to the development of more stable and effective vaccines. Combining these biomaterials with mRNA technology provides a promising framework for future vaccine development. Overall, this review underscores biomaterial-driven antiviral systems as a major frontier in translational medicine with profound implications for global health and pandemic awareness.

## 1. Introduction

**Figure 1:**
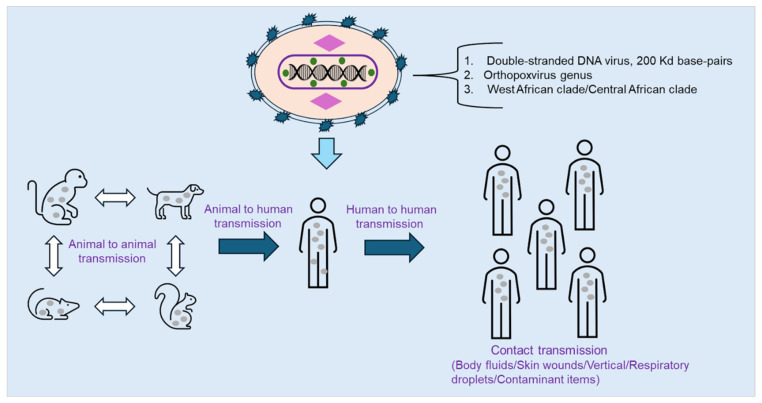
The diagram depicts the MPXV virus, which is a double-stranded DNA virus from the Orthopoxvirus gene, and the transmission mechanistic pathway from infected animals to humans through direct contact or contaminated materials.

The recent rise in Monkeypox virus (MPXV) [1] infection has sparked growing concern about a public health crisis worldwide. Formerly known as a rare zoonotic infection found mainly in parts of Central and West Africa, MPXV has demonstrated its capacity to spread beyond endemic regions [2]. The global outbreak in 2022 underscored the urgent need for a deeper understanding of the virus’s evolution and the development of effective treatment and prevention strategies [3,4]. MPXV can be transmitted through direct contact with an infected animal (body fluids/tissues/meat consumption), an infected person (body fluids/tissue/respiratory droplets), or contaminated materials (Figure 1) [5]. MPXV is a large, double-stranded DNA virus belonging to the *Orthopoxvirus* genus of the *Poxviridae* family, sharing close genetic ties with the smallpox virus. Its genome spans about 200 kilobases and encodes numerous proteins that play roles in replication, immune evasion, and host interaction [6,7].

MPXV is classified into two major genetic clades, the West African (WA) clade and the Central African or Congo Basin (CB) clade, based on genomic variations (5% genetic difference at the whole-genome level) [8,9]. As a double-stranded DNA virus, MPXV has a relatively low mutation rate (10^−6^–10^−7^ mutations per base pair per year) compared to RNA viruses such as SARS-CoV-2 (10^−3^ mutations per base pair per year) [4]. Consequently, the virus accumulates few single-nucleotide polymorphisms (SNPs) over time, indicating limited genomic variability and the sustained efficacy of existing vaccines and antiviral drugs [10]. The World Health Organization (WHO) designated the outbreak a Public Health Emergency of International Concern in July 2022. Though the emergency status ended in May 2023, the outbreak illustrated MPXV’s ability to circulate in global populations, challenging existing containment protocols [11]. Historically, MPXV transmission was primarily animal-to-human, with rodents such as Gambian pouched rats and squirrels acting as reservoirs [12]. The virus was first observed in captive monkeys in 1958, while human infection was first reported in 1970 in the Democratic Republic of Congo [13,14]. Both the 2003 U.S. outbreak and Nigeria’s 2017 outbreak with over 200 confirmed cases emphasized the virus’s potential to establish itself outside traditional regions and the absence of cross-protective immunity [15,16,17].

MPXV mutates slowly, but small genetic changes (SNPs) still occur and create variation. These genetic changes impact the viral replication and spread and alter the immune system. Differences in SNPs between strains benefit the virus in terms of adaptation and survival in diverse environments [16]. Hence, early detection and point mutation(s) in the MPXV sequence may restrict the spread/inhibition of the MPXV gene. Mpox typically presents with both systemic and dermatological features, including early symptoms such as fever, headache, myalgia, fatigue, and notably lymphadenopathy, which helps distinguish it from similar infections. Within a few days, characteristic skin lesions develop and progress through stages such as macules, papules, vesicles, pustules, and finally crusts, commonly affecting the face, extremities, and anogenital region. Pseudomembranous (PM) lesions are relatively uncommon and may occur on mucosal surfaces such as the oropharynx or genital areas, appearing as whitish or yellowish membrane-like patches due to epithelial damage, inflammation, and necrosis, and may be associated with pain, dysphagia, or secondary infections. The reported mortality rate varies depending on the viral clade and outbreak setting, generally ranging from less than 1% in recent outbreaks to approximately 10% in historically reported cases.

To mitigate MPXV’s impact, a combination of genetic and pharmaceutical strategies is being investigated [18,19,20,21]. Gene-targeting technologies such as CRISPR-Cas systems and RNA interference may have the potential to silence essential MPXV genes involved in viral replication and immune evasion [22,23]. Small-molecule inhibitors and antisense oligonucleotides that target specific viral pathways represent promising therapeutic options for managing MPXV infections. In parallel, drugs initially developed for related viruses, such as tecovirimat, are being repurposed as interim treatment options [24]. In addition, appropriate design and inhibition of mRNA expression through antisense treatment and small-molecule-based antiviral treatment will be fruitful for future MPXV treatment [25]. Therefore, our aim in this review is to describe its genetic variability, virus–host interaction, and existing and future approaches for potential MPXV treatments. Unraveling and achieving proper understanding of the genomic variation of MPXV is important for controlling future outbreaks in the coming years.

## 2. Mechanism of MPXV Action

**Figure 2:**
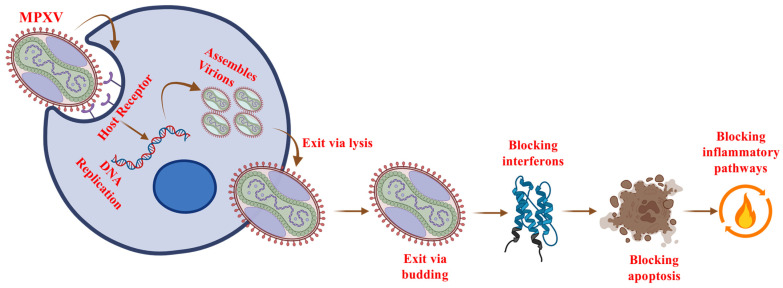
Schematic representation of the Monkeypox virus (MPXV) life cycle. MPXV binds to host cell receptors and enters the cytoplasm, where it replicates its DNA genome. Newly synthesized viral components are assembled into virions, which are then released from the host cell either by lysis or budding. MPXV interferes with host defense mechanisms (i.e., detection of invading viruses and limitation of their replication) by blocking interferon signaling, inhibiting apoptosis, and suppressing inflammatory pathways.

MPXV initiates infection by attaching to host cells through interactions with surface molecules, likely glycosaminoglycans such as heparin-sulfate [26]. MPXV binds to host cells through membrane-associated molecules, particularly glycosaminoglycans (GAGs) such as heparan sulfate and chondroitin sulfate. These are widely present on cell surfaces. GAGs act as initial attachment receptors, allowing the virus to anchor to the host cell. Following attachment, the virus enters the host cell either through direct membrane fusion or via receptor-mediated endocytosis, with subsequent fusion occurring within endosomes [21,27,28,29]. Once inside the cytoplasm, MPXV releases its core, which contains the viral genome and transcription machinery [30]. The transcription machinery of MPXV is the set of viral enzymes and proteins that allow it to copy its genes into RNA inside the host cell. Unlike most viruses, MPXV replicates solely in the cytoplasm of the host cell, utilizing its own enzymes for early gene transcription, effectively bypassing the host’s nucleus [31]. Early viral gene products primarily target the host’s immune system, initiating DNA replication and preparing the cell for subsequent stages of infection. As the infection progresses, intermediate and late genes are expressed, leading to the production of structural proteins essential for viral assembly and replication [32,33,34]. During MPXV infection, viral DNA replication occurs in cytoplasmic “viral factories.” These factories produce immature virions that mature into intracellular mature virions (IMVs). Some IMVs are released by cell lysis, while others become intracellular enveloped virions (IEVs) transported to the cell surface by microtubules, ultimately releasing extracellular enveloped virions (EEVs) for further spread (Figure 2) [35,36]. MPXV employs various immune evasion strategies, including inhibiting interferon signaling, blocking apoptosis, suppressing inflammatory responses, and interfering with antigen presentation pathways [32]. These strategies, mediated by viral proteins, allow MPXV to evade the host’s immune system and prolong infection [28]. Understanding these immune evasion mechanisms is crucial for developing effective antiviral therapies and control strategies [34].

## 3. Biomaterial-Enabled Delivery Platforms

### 3.1. CRISPR-Based Antiviral Approaches

CRISPR-based antiviral approaches for MPXV gene editing are a very promising area of research [37,38]. Since CRISPR-Cas systems have shown great potential in targeting and editing viral genomes, they could theoretically be employed to tackle viral infections like MPXV [39,40]. In a proof-of-concept study, Williamson and colleagues developed a portable, isothermal CRISPR-Cas12a-based diagnostic assay for the detection of MPXV [41].

Wang and colleagues developed a rapid and reliable diagnostic platform, termed MPXVRCC, by integrating recombinase polymerase amplification (RPA) with a CRISPR/Cas-based detection system for the identification of MPXV and differentiation between its two major clades, the Central African (MPXV-CA) and West African (MPXV-WA) lineages [42,43]. Wang and co-workers introduced a one-step CRISPR-based diagnostic system, termed SCOPE (Streamlined CRISPR On-Pod Evaluation), designed for rapid and ultra-sensitive detection of MPXV in field and low-resource settings [44]. In addition, Cas9 is the most widely studied CRISPR protein and creates double-strand breaks in DNA, which can be used to disrupt viral genes, while Cas13, another CRISPR protein, targets RNA after transcription [45,46,47]. However, among the CRISPR-based approaches to combating MPXV, the prime approach is to identify crucial genes of the MPXV genome that are involved in its replication or infectivity to inhibit viral replication by targeting surface proteins and immune evasion proteins [18,48].

**Figure 3:**
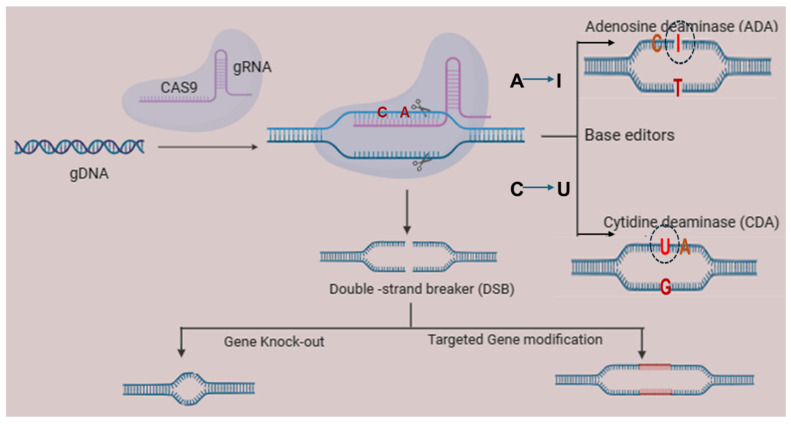
Possible CRISPR approach for targeting the MPXV gene. CRISPR-Cas9 functions as a double-strand DNA cleavage system that enables precise gene targeting and disruption. Base-editor enzymes (ADA and CDA) can perform base modification, which can modify MPXV DNA, particularly inhibiting viral replication and facilitating the development of effective antiviral therapeutics. Herein, we envision that adenosine deaminase (ADA) and cytidine deaminase (CDA) preciously convert adenine (A) to inosine (I) and cytosine (C) to thymine (T) in the MPXV gene. Therefore, the use of these deaminases in a targeted approach will be able to alter the infectious nature of the MPXV gene.

In addition, we propose that a CRISPR-based approach can either knock-down or modify MPXV genomic DNA so that its transcription ability will be destroyed. Furthermore, two base-editing approaches using adenosine deaminase (ADA) or cytosine deaminase (CDA) can be applied to make the gDNA of MPXV non-infectious (Figure 3). It has already been reported that ADA can convert adenosine (A) to inosine (I), while CDA specifically converts cytosine (C) to uracil (U) [34]. Inosine interacts with cytosine, recognizing it like guanosine. Multiple base editing by deaminase enzyme(s) could be a future therapeutic approach for precious, targeted genome modification, particularly in MPXV to make it non-infectious.

### 3.2. Oligonucleotide Therapeutics-Based Antiviral Approaches

**Figure 4:**
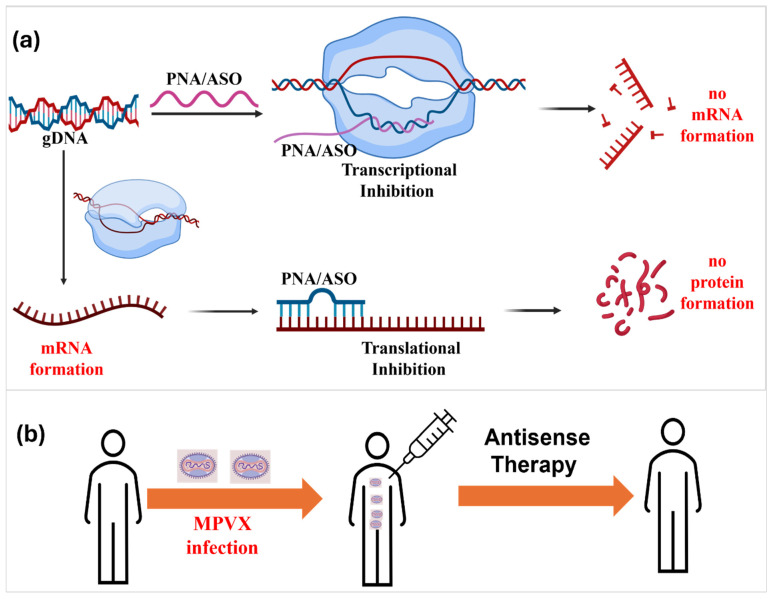
(**a**) Schematic representation of ASO/PNA-mediated suppression of MPXV gene expression. PNAs/ASOs are designed to recognize specific MPXV genetic sequences and bind to viral genomic DNA at promoter regions or to viral mRNAs at untranslated regions (UTRs), thereby blocking transcription and/or translation. This interaction prevents the synthesis of essential viral proteins and disrupts the replication cycle. (**b**) Conceptual overview of antisense-based therapeutic intervention during MPXV infection. Upon viral entry and replication within host cells, targeted delivery of PNAs/ASOs offers a strategic approach to inhibit viral gene expression and limit disease progression.

MPXV releases its double-stranded DNA genome into the cytoplasm of the infected host cell, where the viral DNA-dependent RNA polymerase drives transcription of viral mRNAs necessary for replication [49,50]. Antisense oligonucleotides (ASOs) and peptide nucleic acids (PNAs) are short, synthetic nucleic acid analogues designed to be complementary to specific viral transcripts [51,52,53,54,55]. Upon hybridizing with their target mRNAs, these antisense molecules can sterically block translation or recruit RNase-H to induce selective degradation of the RNA strand [15,36]. By preventing the synthesis of essential viral proteins, ASOs and PNAs disrupt the MPXV life cycle and inhibit the assembly of new virions (Figure 4a). As a result, antisense therapeutics based on PNA/ASO can be a promising antiviral strategy that directly interferes with MPXV viral propagation in the human body [56,57] (Figure 4b). Their high sequence specificity enables precise targeting of viral gene expression while minimizing off-target effects and associated cytotoxicity, making them an attractive platform for antiviral intervention against MPXV and other infectious diseases [58].

Antisense oligonucleotides can target the various virus enzymes responsible for MPXV [59]. The MPXV viral enzyme, Envelope phospholipase F13 protein (F13L), encodes the protein required for virus maturation and release [60]. The protein produced by F13L is involved in the maturation and assembly of new virions. Inhibiting this enzyme could prevent the virus from being properly packaged and released from infected cells. An ASO against F13L mRNA would stop the synthesis of this protein, hindering the virus’s ability to infect new cells. The viral polymerase E9L encodes the RNA polymerase subunit [61]. It is important for transcribing the viral genome into mRNA. The replication of the virus and the production of viral proteins can be stopped through the antisense blocking of the E9L viral polymerase.

### 3.3. RNA Interference-Based Antiviral Approaches

RNA interference (RNAi) has been shown to be a powerful antiviral strategy against MPXV. In an important in vitro study, Alkhalil and colleagues selected twelve conserved MPXV genes that are involved in viral entry, replication, and structural functions [62]. They designed 48 siRNA constructs, four for each gene, to test their effects on viral replication in mammalian cells. Two genes—A6R, which is essential for replication, and E8L, which is important for virus entry—were found to be particularly sensitive. siRNA pools targeting these genes reduced MPXV production by about 65 to 95 percent while causing minimal cytotoxicity. Among these, a single siRNA called siA6-a showed especially strong activity, maintaining potent inhibition for up to seven days after infection at concentrations as low as 10 nM. Additional studies on orthopoxviruses have also shown that siRNAs targeting other essential genes, including D5R, B1R, and G7L, can strongly suppress vaccinia virus and, in some cases, MPXV replication. These siRNAs were effective in both preventive and therapeutic settings, reducing viral replication by more than 70 to 90 percent [63]. These findings support the view that RNAi can be a highly specific, tunable tool for MPXV therapy, especially when combined with delivery technologies (e.g., lipid or polymer nanoparticles) and chemical modifications that enhance stability and reduce off-target effects. However, translation to in vivo settings will require overcoming challenges, including efficient delivery to skin lesions, avoiding immune activation, and ensuring a durable effect without viral escape.

### 3.4. Antiviral Small-Molecule Strategies to Combat MPXV

**Figure 5:**
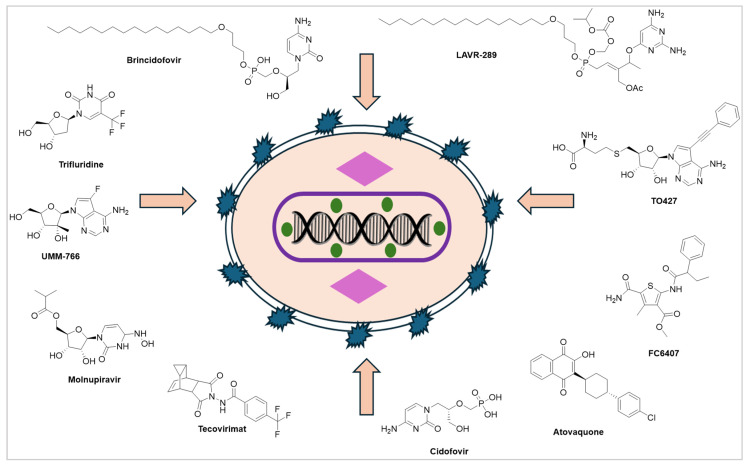
These are the major blockbuster drugs that have shown effectiveness against MPXV and are widely investigated for treatment and control.

There are several existing antiviral drugs available that exhibit potency for the treatment of MPXV (Figure 5). Tecovirimat (TPOXX), originally developed for smallpox, is one of the antiviral drugs that has been shown to reduce the severity and duration of MPXV infection [64]. Tecovirimat works by inhibiting the virus’s ability to spread within the body, thereby limiting the extent of infection and supporting the body’s recovery [65]. This treatment is recommended for high-risk individuals, including those with severe MPXV or underlying health conditions that could complicate the disease [66]. Another antiviral, Cidofovir, has been used off-label for severe cases of MPXV, although its use is less widespread. Cidofovir inhibits viral DNA polymerase, which helps reduce viral replication [67]. However, the use of these antiviral treatments is typically reserved for individuals with more severe disease or for those at high risk of complications [68]. While antiviral treatments can be effective, they must be administered early in the course of infection to maximize their benefit. Additionally, further studies are required to establish standardized protocols for the use of antivirals in MPXV, particularly in varying population demographics and geographical locations [67,69].

**Figure 6:**
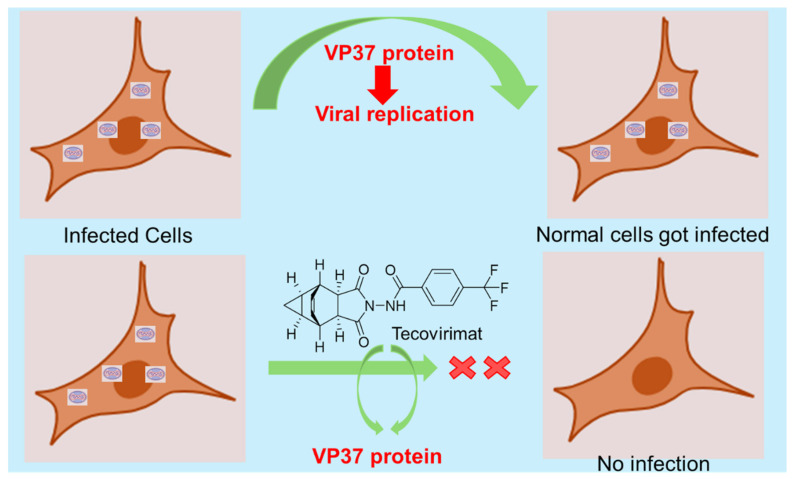
The mechanistic pathway of Tecovirimat, which functions as an antiviral by blocking the replication of the VP37 protein, ultimately stopping the virus from spreading.

The VP37 protein is an important target site for antiviral treatment of MPXV. It is basically a multifunctional protein which impacts its efficient and systematic spread. Antiviral drugs such as tecovirimat, cidofovir, and brincidofovir are used for symptomatic treatment. However, there is no MPXV-specific vaccine available yet. Existing vaccines that are potent against smallpox enhance the immunization rate. Tecovirimat highly inhibits the function of the orthopoxvirus VP37 envelope wrapping protein, a major envelope protein required to produce extracellular virus. Tecovirimat prevents the virus from leaving an infected cell and hinders the spread of the virus within the body (Figure 6). LAVR-289 targets the viral DNA polymerase (insert 2 domain), inhibiting early-stage replication in the cytoplasm. It shows high potency against multiple orthopoxviruses, including the 2022 MPXV clade IIb variant, at nanomolar levels, and demonstrates significant efficacy in animal models by reducing mortality and viral load [25,70]. UMM-766 is a nucleoside analog that inhibits orthopoxvirus replication, including Mpox, and shows effective oral activity in animal models with reduced viral load and protection against severe disease [71]. Trifluridine (TFT) is an FDA-approved topical antiviral used for ocular Mpox infections, where it inhibits viral DNA replication and helps prevent complications, and it is often used alongside Tecovirimat in severe or resistant cases [72]. TO427 is a computationally identified small-molecule inhibitor that targets the VP39 methyltransferase of Mpox, disrupting viral mRNA capping and replication. It shows higher potency than standard inhibitors but remains a preliminary candidate requiring further experimental validation [73]. FC-6407 is an experimental inhibitor of the D4 DNA processivity factor in Mpox, disrupting viral DNA replication by destabilizing the protein, with an *IC*_50_ of about 13.4 μM [25]. Atovaquone is a repurposed antiparasitic drug that inhibits Mpox replication at a post-entry stage by targeting dihydroorotate dehydrogenase (DHODH), thereby reducing viral DNA and virion production, with a higher potency than cidofovir [74]. Atovaquone, mefloquine, and molnupiravir are various small molecules identified as effective against Monkeypox virus (MPXV), exhibiting anti-MPXV activity with 50% inhibitory concentrations ranging from 0.51 to 5.2 μM, surpassing the potency of cidofovir [75,76]. Molnupiravir inhibits viral replication through lethal mutagenesis and shows strong in vitro activity. However, its clinical effectiveness may be limited, and it remains an experimental repurposed candidate without established use for Mpox [74].

### 3.5. Emerging Therapeutic Approaches Targeting MPXV Replication and Entry

**Figure 7:**
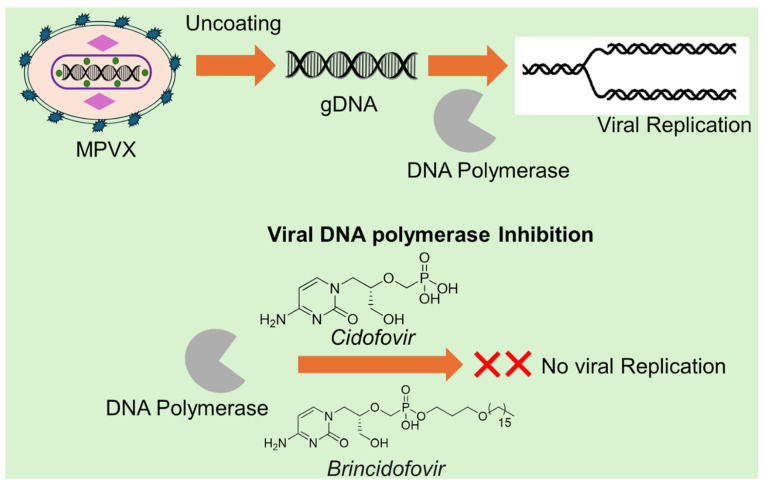
Cidofovir and Brincidofovir are antiviral drugs which compete with the natural nucleotide (dCTP) and lead to premature chain termination followed by inhibition of viral replication. The dual use of the drugs offers a potential treatment for MPXV.

Targeting different stages of the MPXV life cycle offers a promising way to curb infection. One key strategy uses nucleotide analogues like Cidofovir and its lipid-conjugated pro-drug Brincidofovir. These drugs are converted inside infected cells into an active metabolite, cidofovir-diphosphate, which competes with the natural nucleotide (dCTP) at the viral DNA polymerase site and gets incorporated into viral DNA, causing premature chain termination and blocking viral genome replication. Structural and biochemical work confirms this inhibition of the viral polymerase machinery [77,78,79] (Figure 7). Another approach involves small-molecule inhibitors (SMIs) aimed at either viral enzymes or essential host pathways. For example, small-molecule inhibitors against the viral 2′-O-methyltransferase (VP39) enzyme—required for mRNA capping and immune evasion—have shown sub-micromolar to low-micromolar potency in vitro [80,81]. These SMIs have chemical flexibility and potential for use in combination therapy to improve efficacy and reduce resistance. A further complementary strategy uses monoclonal antibodies (mAbs) that neutralize MPXV by binding viral surface glycoproteins needed for entry or cell-to-cell spread. Human mAbs developed against the MPXV A35 glycoprotein have shown strong neutralization in vitro and protection in animal models of MPXV infection. These mAbs act by blocking viral spread and can be engineered to optimize their half-life and effector functions, offering a biologic layer of defense, especially for severe or vaccine-refractory cases [82]. Together, these three modalities, including polymerase-targeting nucleotide analogues, small-molecule enzyme/host-pathway inhibitors, and neutralizing monoclonal antibodies, form a multipronged antiviral arsenal. They act at different phases of the viral life cycle, including replication of viral DNA, post-transcriptional processing with host-cofactor engagement, and viral entry or spread. Using them in combination may reduce the chance of viral escape and boost therapeutic outcomes. However, translation into widespread clinical use remains limited. For example, cidofovir has dose-limiting nephrotoxicity, brincidofovir carries safety concerns, and antibody therapies may be cost-intensive [79].

### 3.6. Molecular Synergism Between Host Lipid Cofactors and Antivirals for Combination Therapy

Structural protein genes of MPXV are located in a conserved central region of its genome and are expressed in different viral forms. The extracellular enveloped virus (EEV) has membrane proteins like C19L, A35R, and B6R, while the intracellular mature virus (IMV) includes proteins such as A29L, M1R, and H3L. The A29L protein is important for viral replication, cell entry, and detection in diagnostic tests [54,55]. The A29L-mediated infection process is thought to involve three main stages, including initial attachment to the host cell surface, activation of membrane fusion by host proteases, and subsequent penetration of the viral particle into the host cell.

E8L is an important surface protein of the IMV and is made of 304 amino acids. According to UniProt (Q8V4Y0), E8L comprises three domains: a virion-exposed region (residues 1–275), a transmembrane domain (residues 276–294), and an intra-virion tail (residues 295–304) [83]. A recent study by Fang et al. identified E8L as a promising antigen for the development of a multivalent mRNA-based vaccine [84].

E8L facilitates viral attachment to host cells by binding to chondroitin sulfate, contributing to the MPXV entry mechanism. MPXV often exploits lipid rafts enriched with negatively charged gangliosides as portals for entry [62]. Fantini et al. mapped the ganglioside-binding domain within E8L using multiparametric analysis, identifying three linear epitopes (residues 43–62, 94–113, and 204–223) that overlap with the ganglioside-binding site [83]. These epitopes were proposed as potential immunogenic targets for vaccine development against MPXV. Such insight could facilitate the design of novel small-molecule inhibitors or enable the repurposing of existing broad-spectrum antivirals to block MPXV entry.

Although no specific antiviral therapy for MPXV has been approved to date, the concept of combination therapy successfully implemented against HIV, hepatitis C, and COVID-19 can be actively explored for MPXV treatment. Understanding the role of host lipid cofactors in MPXV fusion is therefore vital for the development of effective antiviral strategies [85]. Among host-derived antiviral molecules, 25-hydroxycholesterol (25-HC) has demonstrated potent inhibitory activity against a wide range of enveloped viruses and even certain non-enveloped viruses such as human rotavirus [86,87,88].

Beyond its direct antiviral effects, 25-HC functions as a key immunomodulator, regulating inflammatory signaling and cholesterol metabolism to limit viral replication [89]. Additionally, 25-HC modulates the type I interferon (IFN) response, enhancing cellular antiviral states while preventing overactivation that could result in immunopathology [90]. A critical aspect of 25-HC’s immunomodulatory function lies in its regulation of cholesterol metabolism, a process tightly linked to immune activity. 25-HC enhances innate immune defense by mobilizing accessible cholesterol at the cell surface [91]. Furthermore, comparisons of the interaction energies of known antiviral drugs with E8L and A29L along with the cholesterol metabolite 25-HC show that they offer a promising combination therapy strategy for inhibiting MPXV infection.

## 4. Conclusion and Future Perspectives

Biomaterial-based strategies show strong potential for addressing Mpox outbreaks in the near future. Advanced approaches such as CRISPR systems may disrupt viral replication by targeting key genes or applying base editing to render the viral genome noninfectious without inducing double-strand breaks. Antisense oligonucleotides (ASOs), including peptide nucleic acids and short oligonucleotides, can interfere with promoter or untranslated regions of the viral genome, thereby blocking transcription and translation. Antiviral agents such as Tecovirimat and Cidofovir help reduce disease severity and duration, with tecovirimat targeting the VP37 protein, a key component in viral replication. In addition, cooperative interactions between host lipid cofactors and antiviral drugs may further enhance therapeutic outcomes. The integration of biomimetic platforms with nanoparticle-based delivery systems and advanced nanovaccines, including lipid nanoparticle formulations and multivalent protein nanocages, offers improved targeting and efficacy. Collectively, these strategies, including synthetic oligonucleotides, nanoparticles, small-molecule inhibitors, and biomimetic scaffolds, provide biocompatible and potentially low-toxicity solutions that may limit viral transmission and overcome resistance. Furthermore, biomaterials support vaccine development by stabilizing antigens and enhancing immune responses, making them promising tools for managing global health challenges, particularly in resource-limited settings, with further advancements expected through integration with mRNA and gene-editing technologies.

## Data Availability

No new data were created or analyzed in this study. Data sharing is not applicable to this article.

## Abbreviation

List of abbreviations used in the manuscript, along with their full forms and brief definitions for clarity.

MPXV	Monkeypox virus
PNA	Peptide nucleic acid
SNPs	Single-nucleotide polymorphisms
CRISPR	Clustered Regularly Interspaced Short Palindromic Repeats
GAGs	Glycosaminoglycans
IMVs	Intracellular mature virions
IEVs	Intracellular enveloped virions
EEVs	Extracellular enveloped virions
MPXVRCC	Monkeypox virus rapid and reliable diagnostic platform
RPA	Recombinase polymerase amplification
MPXV-CA and MPXV-WA	MPXV Central African and MPXV West African
SCOPE	Streamlined CRISPR On-Pod Evaluation
ADA	Adenosine deaminase
CDA	Cytosine deaminase
ASOs	Antisense oligonucleotides
RNAi	RNA interference
TPOXX	Tecovirimat
SMIs	Small-molecule inhibitors
mAbs	Monoclonal antibodies
25-HC	25-hydroxycholesterol
IFN	Type I interferon
